# Evolving from Morbidity and Mortality to a Case-based Error Reduction Conference: Evidence-based Best Practices from the Council of Emergency Medicine Residency Directors

**DOI:** 10.5811/westjem.2020.7.47583

**Published:** 2020-10-06

**Authors:** Yashwant Chathampally, Benjamin Cooper, David B. Wood, Gregory Tudor, Michael Gottlieb

**Affiliations:** * The University of Texas Health Sciences Center at Houston, Department of Emergency Medicine, Houston, Texas; † Yale University Medical Center, Department of Emergency Medicine, New Haven, Connecticut; ‡ University of Illinois College of Medicine at Peoria/OSF Healthcare, Department of Emergency Medicine, Peoria, Illinois; § Rush University, Medical Center, Department of Emergency Medicine, Chicago, Illinois

## Abstract

Morbidity and mortality conferences are common among emergency medicine residency programs and are an important part of quality improvement initiatives. Here we review the key components of running an effective morbidity and mortality conference with a focus on goals and objectives, case identification and selection, session structure, and case presentation.

## BACKGROUND

Learning from medical errors and near-misses based on retrospective, single-case outcomes is an ubiquitous part of medical training, so much so that morbidity and mortality (M&M) conferences are a required component of graduate medical education in the United States and have been since 1983.^1^ Despite widespread use of the M&M conference, its format remains heterogenous with significant variation between programs.^1^,^2^

The origin of the M&M conference can be traced to the early 20th century when Ernest Codman, a surgeon and outspoken reformer at Massachusetts General Hospital, introduced the end-results system, which employed end-result cards to publicly document individual surgeon’s outcomes.^2^ While this system of blame assignment was met with intense opposition at the time, it largely informed the initial iteration of the M&M conference.^2^ Despite over a century of shared experience with M&M conferences among medical centers, many of the limitations of the primitive M&M conference still exist today. These include haphazard retrospective collection of data, focus on isolated and anecdotal events without consideration of previous similar events, recall bias, lack of meaningful audit, narrow focus on individual performance, lack of systems-based thinking, and lack of multidisciplinary involvement.^3^–^5^

Recently, there has been a shift toward incorporation of quality assurance (QA) and quality improvement (QI) goals and objectives within the framework of the traditional M&M conference.^2^ In this paper, we perform a narrative review of the literature and provide best practice recommendations for goals and objectives, case identification and selection, and the structure and case presentation of M&M conferences. Using the available evidence, these recommendations redefine the conference’s purpose and revise the outdated elements of the traditional M&M conference, including the proposal for a new title to better reflect the goals of the session – case-based error reduction conference (CBERC).

### Critical Appraisal Of The Literature

This article is the fifth in a series of evidence-based best practice reviews from the Council of Residency Directors in Emergency Medicine (CORD) Best Practices Subcommittee.^6^–^10^ A literature search was performed by a medical librarian of databases including ERIC, Embase, CINAHL, Medline, and Web of Science for articles published from inception through February 7, 2019, using combinations of keywords including education level (graduate, medical, internship, house staff, PGY, and residency), conference (or didactics or lecture), and “morbidity and mortality.” Two authors independently screened the resulting papers for relevant articles addressing M&M conference. Additionally, bibliographies were reviewed for applicable references not included in the initial literature search.

The literature search yielded 1199 articles, of which 51 were deemed relevant for inclusion. When there was a paucity of supporting data, recommendations were made based on our consensus opinion and experience. We used the Oxford Centre for Evidence-Based Medicine criteria to provide level and grade of evidence for each statement (Tables 1 and 2).^11^ Prior to submission, the manuscript was reviewed by the entire CORD Best Practices Subcommittee and then posted to the CORD website for two weeks for general feedback and review from the entire CORD community.

## DISCUSSION

### Goals and Objectives

The objectives of M&M conferences vary widely across residency training programs.^1^,^2^,^12^,^13^ Without any established best-practice recommendations and a limited body of robust literature, many conferences operate based on local institutional experience and the potentially limited knowledge of the educators administering the conference. QA is the process of using monitoring systems and retrospective performance analysis to determine whether expected standards are being met. QI is the application of data, including data gathered from QA activities, to improve systems and individual performance. The goal of the application of QA/QI activities is to prevent the occurrence or recurrence of errors through system and process improvement in the interest of delivering better patient care.

The goal of a conference-based or a classroom-setting interaction with medical staff is generally focused on education and information transfer. Historically, M&M conference has sought to improve patient care through education using a case-based format. However, the attempt to combine the QA/QI goals with those of medical education has been a more recent development.^2^ In fact, Gerstein et al noted that the “typical [M&M conference] format has many shortcomings, including lack of understanding of human factors and systems thinking, a narrow focus on individual performance to the exclusion of the contributory team and larger social issues, hindsight bias, and a lack of multidisciplinary integration into a system-wide safety culture.”^4^ In short, traditional M&M conferences lack standardization, structure, and clear objectives. In the era of increased focus on patient safety, individual departments, institutions, and professional organizations have begun to deconstruct the M&M conference with the goal of transforming it into a mechanism to improve healthcare through education and process improvement.^14^

The traditional title, “morbidity and mortality,” implies that the occurrence of an adverse patient outcome is a necessary trigger. This implication contradicts the evolution of QA/QI best practice, which incorporates near-miss error reporting and analysis as the highest yield source of error prevention events.^15^ This ideology is founded on recognizing the importance of learning from errors before they reach the patient.

The foundational objectives of M&M conference reform are twofold. The system-based goal is to review cases to identify process failures and either create new or modify existing department processes to support both patients and clinicians to prevent error recurrence. The individual-based goal is to teach the healthcare team how to identify the individual and environmental factors leading to cognitive errors and address knowledge gaps. Standardized and comprehensive error discussions have not been effectively performed in programs using traditional M&M conference models.^16^,^17^ If done effectively, working towards these goals could help departments standardize care by reducing practice variability. Additionally, a department-wide conference, including nurses, advanced practice providers, and students in addition to faculty and residents, with a system-based error focus would give the care team and individual providers an opportunity to learn from errors, which are most commonly multifactorial, without having to repeat them. Redesigning the traditional M&M conference, in which the providers are exposed and vulnerable when presenting their own cases, to an anonymous shared experience model may improve information transfer while avoiding a punitive or divisive atmosphere.^18^

To avoid the negative emphasis often associated with M&M conference, we propose that M&M conference be renamed to reflect the two goals of classroom-based education and QA/QI as well as the deliberate move away from the perceptions of “shame and blame” associated with them. For the purposes of this article, the term case-based error reduction conference or CBERC will be used to refer to this transition.

**BEST PRACTICE RECOMMENDATIONS - GOALS AND OBJECTIVES**

1. Emergency departments should hold regular case-based error reduction conferences (CBERCs) with a system focus to help standardize care (Level 3a, Grade C).
2. Programs should ensure that their CBERCs reflect sound educational goals and move away from the perceptions of “shame and blame” associated with the traditional M&M conference (Level 5, Grade D).

### Case Identification and Selection

#### A. Incident Identification

An incident is generally defined as any variance that may ultimately represent a potential or experienced error after complete analysis.^19^ The existing literature is sparse with regard to clearly defined processes to identify these potential errors and determine whether they have educational or QA/QI value. Incident identification is understandably the cornerstone of any effective QA/QI process. Given that the healthcare system lacks real-time, third-party oversight, it is dependent on other retroactive mechanisms for identifying errors that result in discernible harm or near-misses. Without a comprehensive incident identification mechanism, determining error prevalence is not possible. Consequently, the QA community must heavily rely upon voluntary reporting and retrospective medical record screening. Voluntary reporting sources include department physicians, nurses, students, and other employees, as well as external referrals from other services, administration, and patients.

The number and completeness of reports are limited by a variety of barriers. These barriers may include lack of clear departmental expectations for reporting; lack of a convenient mechanism for reporting; lack of anonymity for the reporter; feelings of sympathy for or a perceived need to protect colleagues; fear of litigation; fear of retaliation toward the reporter; and a lack of trust in administrative handling of the report.^19^,^20^ A survey of medical and surgical residents suggested that residents find reporting time-consuming and cumbersome, and some expressed fear of repercussions.^21^ All of these factors can lead to the development of an anti-reporting mindset and a departmental culture of non-participation and ineffectual identification of incident-based improvement opportunities. Therefore, it is essential to have departmental and institutional support to create a culture of safety with an emphasis on improvement over blame. One group described a web-based reporting tool used to identify high-yield cases to facilitate reporting and allow for anonymity if desired.^19^,^22^

Generally, the process for incident identification consists of the following three components: 1) standard medical record review of pre-defined screening parameters; 2) provider reporting efforts; and 3) referrals from other service lines. Several emergency departments (ED) employ institutional screens for predefined events that may identify an opportunity for improvement. Examples of screening categories are listed in Table 3.

Most hospitals have the ability to track these events and provide a list of cases for review. Modern electronic health records may also be able to assist with identifying cases. Departmental leadership or a case review committee should review the health records of each screening-identified patient encounter to search for medical errors. The case analysis process for error identification varies widely between institutions. It may be tasked to a single individual or a QA/QI committee. Regardless of who performs the reviews, the reviewers should have ongoing training in QA/QI, so they can continuously apply best practices with a sophisticated understanding of the science and psychology of QA.

It is important these reviews be separate from traditional peer-review committees, as the emphasis is on process failures, as opposed to individual performance. This separation from peer review committees is critical for two reasons. First, prematurely focusing on individual performance may distract the reviewers from subtle system defects that contributed to the issues associated with the identified case. Second, premature critique of individuals can erode the trust of faculty and residents in the overall QA process and further undermine subsequent reporting. In the case that an incident has both process and individual performance concerns, it is incumbent on the QA committee and departmental leadership to ensure they are addressed separately. Interjecting peer-review elements into CBERC will undermine the goals of QA/QI centered conference by distracting from the focus on systems issues and introducing tension and anxiety regarding individual performance.

Predefined screening-based reviews may only identify a small number of the errors within a given department. Thus, other reporting mechanisms should be sought to identify as many potential cases as possible. Table 4 includes a list of other potential sources for case identification.

Ideally, reporting should be anonymous and confidential; however, anonymous reports can be problematic if the report is incomplete or requires further clarification in order for the investigator to sufficiently assess the case for errors. The ability to communicate with the reporter may be invaluable. Therefore, while anonymity should be offered to increase reporting, an emphasis should be placed on providing the reporter’s name to better understand the case components.

#### B. Case-based Evidence Review Conference Case Review and Selection

Several methods of standardized case review may be used and will largely depend on the number of cases and staffing available to investigate.^23^ Berenholtz et al describe using a “defect tool” in their new M&M conference format stating “... to learn from medical incidents and improve patient safety and quality of care, caregivers need to do the following: 1) elicit input from all staff involved in the incident^12^; 2) use a structured framework to investigate all underlying contributing factors; and (3) assign responsibility for management [of process changes] and follow-up on recommendations.”^24^ The exact details of the technique used may be less important than the general incorporation of a standardized methodology as many programs reported perceived improvement with a wide variety of standardized tools. One study found that participants in a surgical program believed that group peer review was substantially less heterogeneous than that of a single individual.^25^ Error analysis by group consensus is likely to yield less concern for variability and misclassification of cases.

Cases should be carefully selected for their value in achieving the goals of process and performance improvement. Selected cases should have a broad educational value such that a meaningful proportion of providers may benefit from the error prevention strategies discussed. Cases that do not have a clear value to the audience-at-large should be avoided and referred to other venues for remediation (eg, peer review committee). Some cases may have broad educational or process improvement value, in addition to isolated concerns for provider competence or professionalism. It is critical to handle these cases in such a way as to accentuate the educational or process improvement points while preventing the other, more personal elements from distracting the participants.

Cases selected should be analyzed diligently to determine the nature of the error and the impact on the patient. Several case analysis tools are available from the QA/QI literature that can be used as part of a standard review process (Table 5).^23^,^24^,^26^–^28^ Gathering accurate information regarding the case details may require iterative feedback from the care team involved in the case in order to generate an accurate description of case details. Feedback should be sought early and in sufficient detail to ensure a high-quality review, as the health record may not fully or accurately reflect the care episode and delays in discussing the case may result in substantial recall bias.^5^ This two-way communication will also improve the ability to provide early feedback to the involved providers and give them an opportunity to clarify events. Subsequently, involved providers should be informed of the committee consensus and plans for anonymous presentation with the opportunity to clarify events at the CBERC if they desire. In addition to improving the quality of case reviews, two-way communication supports the care team members, so that they do not feel attacked or misrepresented. The process should be inclusive, transparent, and conducted with sensitivity, respecting both the patient and family, as well as the care team members.

**BEST PRACTICE RECOMMENDATIONS – CASE IDENTIFICATION AND SELECTION**

1. Incident identification should occur via web-based reporting and/or institutional screens for predetermined events (Level 4, Grade C).
2. Case analysis should be separate from peer review so as not to distract from the focus on system issues (Level 5, Grade D).
3. Error analysis should be performed by group review using a standardized methodology (Level 3a, Grade B).
4. Feedback from involved providers should be sought early and in detail as the health record may not accurately reflect the care episode and to avoid recall bias (Level 3b, Grade C).
5. Cases should be carefully selected for their value in achieving the goals of process and performance improvement (Level 5, Grade D).

### Structure and Case Presentation

Programs that instituted standardized, structured approaches to CBERC with a focus on system-based errors found that the resident perceptions of the conference were consistently more positive than prior to the structured apporach.^28^–^31^ Although there is not yet a consensus on the ideal format for achieving the objectives of CBERC, several elements and models have been described that have demonstrated improvement in the experience and the ability to translate error analysis to meaningful QA/QI initiatives.^32^–^34^ Studies have consistently demonstrated that the perceptions regarding standardizing error analysis are positive when compared to previous less structured conferences within a given department.^18^,^31^,^34^–^38^

Table 6 includes a list of elements associated with a successful CBERC based upon a study by Mitchell et al. This intervention was associated with increased faculty satisfaction, improved presentations, and greater retention of learning points by the residents.^32^ In addition to those listed in Table 6, the situation, background, assessment/analysis, and review of literature with recommendations (SBAR) format has also been proposed as a useful format.^32^,^39^

The case presentation should have a standard format to organize the content in a way that is easy to follow and comprehend.^40^,^41^ The error description and classification should be based on a standard taxonomy. Additionally, the presentation should include best practice educational elements that optimize information delivery and retention for the audience.

In a survey of 33 residency training programs, the majority of residents stated that they believed that M&M conference should be non-punitive (72%), educational (87%), and contribute to a culture of safety (78%); however, almost half reported no feedback from cases discussed in the M&M conference, and three-quarters reported no debriefing.^42^ Incorporating structured feedback into an organized process will likely further enhance information transfer and participant satisfaction.^42^,^43^ Therefore, it is important to ensure that there are clear goals and take-home points.

#### A. Anonymity and Immunity

Any CBERC should be conducted under the guidelines and protection of the institution’s QA/QI umbrella. The Healthcare Improvement Act of 1986 (Title 42 of the United States Code, Sections 11101 – 11152) extended state-level immunity for quality assurance and “performance appraisal” activities to the federal law. Unfortunately, these laws do not pertain to medical boards and other licensing organizations, which may request QA/QI records to inform a given investigation. In addition to national and local legal protections, it is critical to clearly and overtly establish that all participants in a departmental CBERC process are protected from retaliation from the department leadership, as well as other members of the medical staff and care team. The CBERC leaders should make every effort to preserve anonymity within the structure of the case presentation to reduce fear of reporting and erosion of trust.^12^,^20^

#### B. Moderators, Statement of CBERC Objectives and Rules of Conduct

The evolution of retrospective, case outcomes analysis for the purpose of QA/QI has resulted in the transformation of a traditionally provider- or institution-centered effort to one that is patient centered. For this reason, the objectives and guidelines for conduct should be explicitly stated at the outset of each CBERC, reminding the participants of the expectations for a collegial and productive learning environment. In addition to the opening guidance, the assigned moderator should ensure that the tone and content of discourse throughout the presentation continues to meet with the expressed goals and rules of conduct.

Many of the participants in a structured CBERC as described here may not have had any substantial QA/QI training. Orienting new participants on an annual basis to the philosophy and design of CBERC may help prepare participants to understand the goals and offer insights and reminders around the principle of a culture of safety. Patel and colleagues surveyed residents after an introductory lecture series on morbidity and mortality concepts. They demonstrated that residents improved their knowledge of M&M conference and felt more comfortable presenting after the training.^44^,^45^ Additionally, both faculty and resident moderators should be trained to present the case findings in a fair-minded, objective manner and to facilitate discussion while preventing participants from deviating from the stated goals to focus on more personal agendas.

Given the general emotional impact of some inevitable performance critique in the context of errors and patient harm, it is important to not take a judgmental, overly prescriptive, or authoritarian tone. Such an approach risks reinforcing negative experiences or perceptions with CBERC and medical errors. In addition, it may be helpful to reinforce available local support resources like employee assistance programs. Every effort should be made to engage the audience in both the error analysis discussion, as well as the error remediation or prevention components of the presentation. The moderators should also be trained to use nonjudgmental language reinforcing positive themes such as patient-centered focus, teamwork, collegiality, and improving together. Ending the conference on a positive note may also help relieve tension and promote engagement. For example, ending the meeting by recognizing outstanding resident performances may help alleviate concerns of focusing solely on a handful of mistakes rather than the excellent care that constitutes the majority of care encounters.

#### C. Case Presentation

The core of the presentation will be the series of clinical events from the case in question. A standardized format should be employed, and the level of detail and timeline should be consistent with the error identified and the preceding contributing events. The moderators should have access to all the clinical data if the discourse raises unanticipated questions, but the presentation itself should be succinct. The CBERC organizers should attempt to find a balance between excessive brevity and an exhaustive inclusion of every clinical detail. The following three elements leading up to the error should be included: 1) clinical data; 2) ancillary data; and 3) timeline.

Accurately recounting the case in a classroom format may not perfectly capture all of the dynamics encountered in the actual clinical environment, but it may be enhanced in several ways. First, by recreating the clinical experience of the providers involved in the case, with pauses for audience participation, the audience can appreciate the challenges the providers faced, as well as assess their own knowledge anonymously. This encourages empathy rather than allowing assumptions regarding the likelihood of making the same or similar error.

Audience-response poll questions could provide an important, low-pressure, self-assessment opportunity for participants. For example, a question requiring interpretation of an electrocardiogram may provide information regarding a knowledge gap that can be addressed by the participant, as well as the education leadership. Second, visualizing real-time data from audience members may help the CBERC organizers gain insight into process or knowledge gaps. For example, a question regarding an existing department policy may provide valuable information regarding what proportion of the audience is familiar with the policy. This practice of interpolating questions promotes retrieval, a critically important task for learning,^46^,^47^ and has been shown to increase learners’ ability to sustain attention, encourage task-relevant notetaking, and improve learning and enjoyment.^48^–^50^

Another method of increasing engagement is the use of simulation. Vozenilik described the use of previously recorded simulations based on M&M conference cases in which audience participants view the recording and make decisions within the context of a patient encounter.^44^ This would allow participants to experience the scenario in real time and identify additional areas for improvement.

#### D. Discussion and Error Classification

Once the outcome of the case is disclosed to the audience, the error should be categorized based on the impact to the patient. If the error reached the patient and contributed to a poor outcome, then the degree of patient harm is classified as either minor or major depending on the outcome. The harm can be further classified as physical, psycho-emotional, patient inconvenience, or financial. If the error made no discernible impact on outcome, then it can be classified as a near-miss. In addition to patient impact, the error can be classified by the impact to the department or institution including resource stewardship. For example, an error resulting in an avoidable increased length of stay represents a resource loss in the form of monopolizing a bed, which may impact ED throughput.

Historically, M&M conference has focused on the most serious outcomes rather than minor events or near-misses as is evidenced by the traditional name of the conference. Special effort should be made to explain the value of errors that result in near-misses or have a minor impact on the patient if these cases add educational or process value.^15^,^51^ As departments transition from the traditional morbidity and mortality model to a more QA/QI-focused process, there may be a reluctance to include cases without any discernible patient harm, as the participants have been habitualized to discussing cases with the most severe outcomes. The participants should understand that the greatest improvement value in retrospective error analysis lies in near-miss and minor harm cases because they represent the vast majority of potential cases.

Through the use an existing taxonomy, the error should be classified by type. Using standard language in assessing the error will help the CBERC organizers create a consistent, uniform approach, which can facilitate tracking and trending of the errors in a department error registry or database. Reviewing the various taxonomies available is beyond the scope of this article, but one such taxonomy that has been used successfully by the authors is shown in Table 7.

In discussing and classifying errors, there is a natural tendency to divert focus from patient care to medicolegal risk for the provider or the institution. Although CBERC may lend itself to risk management-centered teaching, the CBERC organizers and moderators should be careful to maintain a patient-centered focus. It is certainly reasonable to capitalize on risk-management teaching moments as they arise naturally, but the dominant theme should not stray from patient care to legal risks as this may erode the foundational paradigms surrounding QA/QI. Other settings like a “mock trial” format may serve as a better mechanism for the “teaching to the tort” model.^53^

#### E. Case Closure and Error Reduction Strategies

Aaronson et al found that despite having process improvement objectives, many programs have no feedback or follow-up process by which to effect changes.^12^ Siegel et al performed a national survey with similar results.^13^,^54^ By describing the errors, the sequence of events, and contributing factors that led to the error, the presenter will be able to make recommendations regarding error prevention. Once the contributing factors and root causes have been dichotomized into remediable vs non-remediable, the moderators and CBERC organizers can suggest mechanisms to improve the remediable factors. It is critical to engage the audience in this process in order to take advantage of brainstorming in a group. This will facilitate a greater understanding of the issues, broaden list of potential solutions, and increase support for proposed solutions.

In the interest of high-quality information transfer and retention, the core lessons for each case should be reinforced at least twice during the presentation. Examples of techniques include clearly declaring a “take home message” both verbally and visually or rapid question-answer sequences that test the audience recall. Meenakshisundaram et al reported consistent improvement in knowledge using pre- and post- M&M conference questions following case presentations, with knowledge retention maintained at three months.^55^ Table 8 provides an overview of a CBERC presentation.

#### F. Post-case-based error reduction conference debrief to generate consensus on error reduction strategies and QI projects

Incorporating and applying QA/QI principles will create a natural transition from error identification to error reduction in the form of QI projects intended to change processes.^29^ A group of organizers, along with the moderators, should convene to review the cases and make sure there is consensus regarding the error types and the care improvement strategies generated in the conference. As previously mentioned, vetting cases in a group dynamic is more likely to be viewed as fair and transparent.^25^ Maintaining databases for both error types and reduction strategies can help identify departmental trends, as well as provide ideas for future QI work.^29^,^56^ There should also be a feedback mechanism regarding what went well and what areas need improvement with regard to the presentation style or content.

**BEST PRACTICE RECOMMENDATIONS – STRUCTURE AND CASE PRESENTATION**

1. Programs should institute a standardized, structured, and systems-based approach to case presentation (Level 3a, Grade B).
2. Error classification should be based on standard error taxonomy (Level 5, Grade D).
3. CBERC should make every effort to preserve anonymity within the structure of case presentation to reduce fear of reporting and erosion of trust (Level 3a, Grade B).
4. The educational and safety-promoting focus should be clearly and consistently reinforced at the onset of each CBERC (Level 4, Grade C).
5. The periodic use of polling response systems can provide a simulated environment to stimulate learning (Level 2a, Grade B).
6. CBERC moderators should engage the audience in the process of error prevention for errors that are remediable to take advantage of the group dynamic (Level 5, Grade D).
7. A group of organizers should convene post-CBERC to gain consensus on error types and improvement strategies generated in the conference, facilitating the formation of QI projects (Level 3b, Grade C).

## LIMITATIONS

There are several limitations to consider with regard to this review. First, it is possible that we may have missed some relevant articles. However, an experienced medical librarian conducted the search using a broad search strategy across multiple databases. We also reviewed the bibliographies of all included articles, contacted topic experts, and underwent pre-submission peer review by the entire CORD community. Additionally, some areas did not have EM-specific data available. In these cases, relevant data from other specialties and fields was incorporated where appropriate. When limited evidence was available, recommendations were based upon expert consensus.

## CONCLUSION

As quality- and safety-related programs evolve, there is an increasing recognition of the importance of analyzing near-misses in healthcare error reduction. The classic M&M conference model implies that a bad outcome is necessary prior to error analysis and remediation. The vast majority of errors relate to near-misses and therefore represent the greatest opportunity to improve processes. Additionally, the M&M conference title is fraught with potential for negativity and apprehension due to the often punitive and trial-like nature of traditional conferences. Therefore, we recommend a new title – case-based error reduction conference. We recommend building a culture of safety in which leaders create a non-punitive structure that focuses on systems issues and avoids individual “blame and shame” tactics.

Other structural elements likely to be successful include transparent incident reporting, multidisciplinary involvement, anonymity whenever possible, case selection for broad educational value, audience participation, and quality improvement. To maximize the educational value of CBERC, audience members should actively participate, central concepts should be recapitulated, and learners should be encouraged to debrief on error reduction strategies and QI projects. This should be conducted in a carefully guarded educational safe space designed to protect patients and providers.

## Figures and Tables

**Table 1:** Oxford Centre for Evidence-Based Medicine criteria.^11^

Level of evidence	
1a.	Systematic review of homogenous RCTs
1b.	Individual RCT
2a.	Systematic review of homogenous cohort studies
2b.	Individual cohort study or a low-quality RCT*
3a.	Systematic review of homogenous case-control studies
3b.	Individual case-control study**
4.	Case series or low-quality cohort or case-control study***
5.	Expert opinion

*RCT*, randomized controlled trial.

* Defined as <80% follow up;

** includes survey studies;

*** defined as studies without clearly defined study groups.

**Table 2:** Oxford Centre for Evidence-Based Medicine grades of recommendation.^11^

Grades of recommendation	
A.	Consistent level 1 studies
B.	Consistent level 2 or 3 studies or extrapolations* from level 1 studies
C.	Level 4 studies or extrapolations* from level 2 or 3 studies
D.	Level 5 evidence or troublingly inconsistent or inconclusive studies of any level

* Extrapolations refer to data used in a situation that has potentially clinically important differences than the original study situation.

**Table 3:** Examples of screening categories for potential medical error.

Return to ED within 48–72 hours with admission
Death in the ED
Death within 3 days of hospitalization
Rapid Response Team activation with escalation of care within 12 hours of hospital admission

*ED*, emergency department.

**Table 4:** Potential sources for case identification with regard to medical error incidents.

Institutional or departmental reporting registries
Feedback from other services
Solicitation from department leadership
Self-reporting
Institution-based standard quality reviews
Patient complaints
Medical staff reporting

**Table 5:** Select case analysis tools.

Tool	Components	Advantages	Limitations
Defect tool^24^	Identify a clinical or operational event that should “never happen again”	Elicits input from all staff involved Incorporates structured framework to investigate all underlying contributing factors Assigns responsibility for management and follow-up	Difficult to find experienced mentors Difficult to curtail enthusiasm regarding widespread system issues and limit project “scope-creep”(ie, shifting the focus from the primary process to a different, partially related process) Difficult to evaluate efficacy of interventions for “rare” errors
Ishikawa (fishbone) diagram^23^,^26^	Include people, procedures, equipment, environment, policy, and other	Uses an approach similar to root-cause analysis Uses a standardized process improvement tool	May need to add a category reflecting “cognitive errors” Usually only one element of a larger analysis
Mayo Clinic 6-step audit^27^	Interview all parties and use a QI tool (eg, fishbone, mind map) for root-cause analysis Determine overall cost and system issue contributing to outcome Propose system level intervention and prioritization	Meaningfully contributes to institutional QI initiative Creates a change in the culture of M&M conference away from “shame and blame”	Requires larger institutional buy-in May involve larger audiences/groups
Mind map^23^,^28^	Use diagram in which the central box represents the adverse outcome or problem Extend links outward in all directions as contributing factors	Cross-links factors on periphery that may have interactions and associations	May need more contextual institutional data Can become large and difficult to interpret for linear thinkers
Vanderbilt Structured Morbidity and Mortality Improvement (MMI) conference^26^	Include all deaths, patient injuries with prolonged or permanent damage, and near-miss (selected by MMI Task Force)	Selects cases with the potential for issues that are system-wide, multi-departmental, or involve more than one patient care population Has a fixed format, reports on progress from prior conferences Includes ACGME Core Competencies	Requires larger institutional buy-in May involve larger audiences/groups

*ACGME*, Accreditation Council for Graduate Medical Education; *QI*, quality improvement; *M&M*, morbidity and mortality.

**Table 6:** Key elements of successful morbidity and mortality conferences.

Making resident and faculty attendance mandatory
Decreasing defensiveness and blame
Improving the efficacy of the case presentations
Using slides
Using radiographic images
Focusing on analysis of error
Integrating evidence-based literature into the case discussion
Providing educational points related to the complication
Encouraging audience participation in the process
Allowing for a consensus to be met with respect to analysis of the cases presented
Having a moderator facilitate the conference
Fostering multidisciplinary involvement

Adapted from Mitchell et al 2013.^32^

**Table 7:** Example of an error taxonomy system.^52^

System/process error	Non-remediable factors	Cognitive factors
Equipment failure	Atypical presentation	Faulty data gathering
High workload	Complicated medical history	Faulty information processing
Inadequate handoff	Language barrier	Faulty information verification
Inefficient process	Limited ability to provide history	Faulty knowledge
Insufficient resources	Patient body habitus	Other
Interruptions	Patient non-adherence	
Non-handoff communication error	Psychiatric issues	
Poor equipment usability	Rare disease	
Supervision failure	Other	
Other		

**Table 8:** Proposed order of case-based error reduction conference presentation.

CBERC order of presentation	Comments or examples
1. Statement of objectives and guidelines for conduct	Example: “The information discussed in CBERC is protected and should not be discussed in forums outside hospital-designated QA activities. The objectives of CBERC are intended to improve patient care through the identification, analysis and remediation of medical errors in a collegial, non-punitive forum. Participants are asked to refrain from unprofessional conduct including the use of any accusatory or inflammatory language that may be construed as targeting, intimidation or shaming.”
2. Case presentation	Provide only data available to the provider at specific timeline intervals.
3. Audience response poll	It is often helpful to poll the audience when a critical juncture in the case presentation is reached. For example, after displaying laboratory values revealing hyponatremia for a patient in status epilepticus, a multiple-choice question regarding the next most appropriate step in management may help identify knowledge gaps.
4. Outcome	Reveal the case outcome.
5. Discussion and error classification	Allow for audience discussion, classify the error, and summarize the core lesson. Repeat steps 2 through 5 until all selected cases have been presented.
6. Kudos	We suggest ending the conference on a positive note to relieve tension. This can be achieved by recognizing outstanding performance at the end of every CBERC.

*CBERC*, case-based error reduction conference; *QA*, quality assurance.
